# Epidemiology of ischemic stroke and hemorrhagic stroke in venoarterial extracorporeal membrane oxygenation

**DOI:** 10.1186/s13054-023-04707-z

**Published:** 2023-11-09

**Authors:** Jaeho Hwang, Andrew Kalra, Benjamin L. Shou, Glenn Whitman, Christopher Wilcox, Daniel Brodie, Akram M. Zaaqoq, Roberto Lorusso, Ken Uchino, Sung-Min Cho

**Affiliations:** 1 Department of Neurology, The Johns Hopkins Hospital, Baltimore, MD USA; 2 Division of Cardiac Surgery, Department of Surgery, The Johns Hopkins Hospital, Baltimore, MD USA; 3 Division of Critical Care, Department of Medicine, Mercy Hospital of Buffalo, Buffalo, NY USA; 4 Department of Medicine, The Johns Hopkins University School of Medicine, Baltimore, MD USA; 5 Department of Anesthesiology, Division of Critical Care, University of Virginia, Charlottesville, VA USA; 6 Cardiothoracic Surgery Department, Heart and Vascular Centre, Maastricht University Medical Centre, and Cardiovascular Research Institute Maastricht (CARIM), Maastricht, The Netherlands; 7 Cerebrovascular Center, Neurological Institute, Cleveland Clinic Lerner College of Medicine of Case Western Reserve University, Cleveland, OH USA; 8 Division of Neurosciences Critical Care, Departments of Neurology, Neurosurgery, Anesthesiology, Critical Care Medicine, The Johns Hopkins Hospital, Baltimore, MD USA

**Keywords:** Venoarterial ECMO, Stroke, Mortality, Trend

## Abstract

**Background:**

While venoarterial extracorporeal membrane oxygenation (V-A ECMO) provides lifesaving support for cardiopulmonary failure, complications may increase mortality, with few studies focusing on ischemic/hemorrhagic stroke. We aimed to determine the trends and associations of stroke incidence and mortality, and their risk factors, including the effects of annual case volumes of ECMO centers.

**Methods:**

Retrospective analysis was performed on the Extracorporeal Life Support Organization (ELSO) registry, including adult V-A ECMO patients from 534 international centers between 2012 and 2021, excluding extracorporeal cardiopulmonary resuscitation. Temporal trend analyses were performed for stroke incidence and mortality. Univariate testing, multivariable regression, and survival analysis were used to evaluate the associations of stroke, 90-day mortality, and impact of annual center volume.

**Results:**

Of 33,041 patients, 20,297 had mortality data, and 12,327 were included in the logistic regression. Between 2012 and 2021, ischemic stroke incidence increased (*p* < 0.0001), hemorrhagic stroke incidence remained stable, and overall 90-day mortality declined (*p* < 0.0001). Higher 24-h PaO_2_ and greater decrease between pre-ECMO PaCO_2_ and post-cannulation 24-h PaCO_2_ were associated with greater ischemic stroke incidence, while annual case volume was not. Ischemic/hemorrhagic strokes were associated with increased 90-day mortality (both *p* < 0.0001), while higher annual case volume was associated with lower 90-day mortality (*p* = 0.001). Hazard of death was highest in the first several days of V-A ECMO.

**Conclusion:**

In V-A ECMO patients between 2012 and 2021, 90-day mortality decreased, while ischemic stroke incidence increased. ELSO centers with higher annual case volumes had lower mortality, but were not associated with ischemic/hemorrhagic stroke incidence. Both ischemic/hemorrhagic strokes were associated with increased mortality.

**Supplementary Information:**

The online version contains supplementary material available at 10.1186/s13054-023-04707-z.

## Background

Venoarterial (V-A) extracorporeal membrane oxygenation (ECMO) is a form of temporary life support for patients with refractory cardio-circulatory or cardiopulmonary failure. It operates by extracting de-oxygenated blood from the patients’ venous system, and reinfusing oxygenated blood into the patients’ arterial system with adequate pressure. However, V-A ECMO support is not without risk, as an increasing number of studies throughout the years have reported on various neurological complications, which in turn are associated with higher mortality [1, 2]. Very few studies focusing specifically on the incidence of ischemic and/or hemorrhagic stroke as a complication of V-A ECMO, excluding extracorporeal cardiopulmonary resuscitation (ECPR) [3, 4]. Additionally, prior ECMO studies in the pediatric population revealed that ECMO centers with lower case volumes had higher mortality, though such studies in the adult ECMO patient population are more limited [5, 6]. Therefore, a robust, large-scale epidemiological study is needed to explore the incidence and outcomes related to ischemic and hemorrhagic stroke in adult patients supported with V-A ECMO.

We sought to characterize the trends regarding ischemic and hemorrhagic strokes across time and ECMO center volume, as well as the effects of stroke on mortality adjusting for known risk factors, with the aim to (1) determine the incidence and trend of ischemic strokes, hemorrhagic strokes, and mortality over the past 10 years; (2) determine the association between the case volume per year of ECMO centers and incidence of ischemic stroke, hemorrhagic stroke, or mortality; and 3) determine the association between mortality and ischemic or hemorrhage stroke.

## Methods

### Study design and population

The Extracorporeal Life Support Organization (ELSO) registry is an international voluntary database that collects information on usage, indications, complications, and outcomes of ECMO support in adults and children from 534 centers worldwide currently. The registry collects data related to patient demographics, clinical characteristics, pre-ECMO conditions, hemodynamic and laboratory values before and during ECMO support, complications that occur during ECMO support, and outcomes such as survival at the time of hospital discharge. Diagnosis and medical history are reported according to the *International Classification of Diseases, 9th Revision* (ICD-9) and *10th Revision* (ICD-10) codes.

This study was a retrospective analysis of the ELSO registry database from 2012 to 2021. Inclusion criteria consisted of adult patients (≥ 18 years) who underwent V-A ECMO support for either cardiac or respiratory failure, and whose ischemic/hemorrhagic stroke data were available. Exclusion criteria consisted of patients whose ECMO was later converted to different modes. The period of 2012–2021 was chosen to assess the current, modern practices of ECMO support over the past decade. Pediatric patients were excluded, as their pathophysiology of conditions requiring ECMO often differs considerably from those in adults. ECPR and venovenous ECMO were also excluded as their risk factors and mechanisms of acute brain injury differ from those of V-A ECMO. This study was approved by the local institutional review board.

### Data collection and definitions

For all included patients, the following data were collected from the ELSO registry: patient demographics, ECMO characteristics, pre- and post-ECMO cannulation laboratory values and hemodynamics, on-ECMO complications and their reported timings, including ischemic stroke, hemorrhagic stroke, and status at hospital discharge. For the variable of ischemic stroke, the ELSO registry terms “CNS diffuse ischemia,” defined as computed tomography (CT) or magnetic resonance imaging (MRI) demonstrating diffuse ischemic changes, and “CNS infarction,” defined as CT or ultrasound or MRI demonstrating localized ischemic change, were combined. For the variable of hemorrhagic stroke, three ELSO registry terms were combined: “CNS hemorrhage,” which was discontinued in 2018, as well as “intra/extra parenchymal CNS hemorrhage” and “intraventricular CNS hemorrhage,” which were newly introduced in 2018.

### Outcomes

Primary outcome was defined as 90-day mortality, while secondary outcome was defined as 30-day mortality.

### Statistical analysis

Continuous variables were reported as medians with interquartile range (IQR). Categorical variables were reported as frequencies with percentages. For the patients’ ages, the ELSO registry records the data as a continuous variable with the exception of the age group “80 or older.” In this setting, the ages of patients “80 or older” were changed to 80, so that age could still be analyzed as a continuous variable. To assess the differences in the demographic information and ECMO-related variables between patients with and without strokes, t test was used to compare the means of continuous variables, while *χ*^2^ test was used to compare proportions with each subgroup sample size greater or equal to 5, or Fisher’s exact test in cases of subgroup sample size less than 5.

The Cochrane-Armitage test and Poisson regression were used to evaluate the trends over time (Aim 1). Logistic regression models were created to identify risk factors associated with on-ECMO complications of ischemic and hemorrhagic strokes, with independent variables including ELSO center volume per year (Aim 2). Kaplan–Meier curves and hazard function curves were generated, and log-rank tests were performed to evaluate the risk factors associated with 90-day and 30-day mortality (Aim 3). Cox-regression analysis was also considered initially, however, ultimately unable to be performed, as the test for the proportional hazard assumption based on the Schoenfeld residuals revealed that this requirement was not met. Instead, multivariable logistic regression models were generated with the dependent variables of 90-day (primary outcome) or 30-day mortality (secondary outcome).

Independent covariates chosen a priori consisted of patient demographics including age and sex; center case volume per year; central cannulation site; pre-ECMO blood gas values of arterial oxygen pressure (PaO_2_), arterial carbon dioxide pressure (PaCO_2_), and pH; post-cannulation 24-h blood gas values of PaO_2_ and PaCO_2_; post-cannulation 24-h blood pump flow rate; ECMO duration; and on-ECMO complications, including ischemic and/or hemorrhagic stroke, brain death, gastrointestinal hemorrhage, cardiac arrhythmia, pump failure, moderate-to-severe hemolysis (peak plasma hemoglobin > 50 mg/dL or at least one occurrence of > 500 mg/L during ECMO, sustained for ≥ 2 days), the requirement of neurosurgical intervention, renal replacement therapy, and/or cardiopulmonary bypass. These specific variables were chosen based on their medical relevance and the clinically hypothesized significance of their associations with mortality. Additionally, the ELSO definitions of blood gas-related variables were updated in 2017 and 2018, such that pre-ECMO PaO_2_ and PaCO_2_ were changed from “worst” values to “closest to” ECMO start time, while 24-h PaO_2_, 24-h PaCO_2_, and 24-h blood pump flow rate were changed from “best” values to “closest to 24-h” of ECMO. As a result, ΔPaCO_2_ was calculated only for patients who underwent ECMO in 2018 or later. Similarly, in 2018, the on-ECMO complication of brain death was more clearly defined, and the variable of neurosurgical interventions was added to the ELSO registry. As a result, the logistic regression models, which included PaO_2_, PaCO_2_, brain death, and neurosurgical interventions as independent variables, were generated only with patient data reported from 2018 or later. Bonferroni correction was used to account for the 58 univariate statistical tests performed, resulting in the change of threshold significance from *p* < 0.05 to *p* < 0.00086 for the univariate tests. Finally, the Pearson correlation coefficient was calculated to evaluate the association between 24-h PaO_2_ and 24-h blood pump flow rate. None of these variables had missing that exceeded 40%. All statistical analyses were performed using R Studio (Version 2023.06.2, www.r-project.org).

## Results

Overall, 34,734 V-A ECMO patients were queried from the 2012 to 2021 ELSO database. Of those, 1693 patients were later converted to different modes of ECMO, and thus excluded from analysis, resulting in 33,041 patients. 20,297 had complete mortality data, of which 12,327 were from 2018 to 2021 (Additional file 1). Table 1 summarizes the variables related to patient demographics, ECMO, and mortality. Among the patients with complete mortality data, 96% (*n* = 19,389) had neither ischemic nor hemorrhagic stroke, 3% (*n* = 659) developed ischemic strokes, 1% (*n* = 287) developed hemorrhagic strokes, and 0.2% (*n* = 38) developed both ischemic and hemorrhagic strokes during ECMO support. The median detection timing was 3.04 days for ischemic strokes and 3.79 days for hemorrhagic strokes since ECMO cannulation. The 90-day mortality was higher for patients with ischemic strokes than those without (65% vs 36%, respectively, *p* < 0.0001, *χ*^2^ test). Similarly, the 90-day mortality was higher for patients with hemorrhagic strokes than those without (72% vs 37%, respectively, *p* < 0.0001, *χ*^2^ test). Many ECMO-related variables were found to be significantly different between patients who developed ischemic or hemorrhagic stroke and those who did not (Table 1). Notably, patients who developed ischemic stroke also experienced other on-ECMO complications more often than those who did not develop ischemic stroke, including longer duration of ECMO support (*p* < 0.0001, t test), as well as more neurosurgical interventions (*p* < 0.0001, *χ*^2^ test), brain death (*p* < 0.0001, *χ*^2^ test), cardiac arrhythmias (*p* < 0.0001, *χ*^2^ test), gastrointestinal hemorrhage (*p* < 0.0001, *χ*^2^ test), moderate-to-severe hemolysis (*p* < 0.0001, *χ*^2^ test), and renal replacement therapy (*p* < 0.0001, *χ*^2^ test). This was similar to the cases of patients who developed hemorrhagic strokes compared to those who did not, including longer duration of ECMO support (*p* < 0.0001, t-test), as well as more neurosurgical interventions (*p* < 0.0001, *χ*^2^ test), brain death (*p* < 0.0001, *χ*^2^ test), cardiac arrhythmias (*p* = 0.0002, *χ*^2^ test), gastrointestinal hemorrhage (*p* < 0.0001, *χ*^2^ test), and renal replacement therapy (*p* = 0.0002, *χ*^2^ test).

**Table 1:** Patient Demographics and ECMO Variables for ECMO-associated Ischemic or Hemorrhagic Stroke

Variable	Ischemic stroke (*n* = 659)	No ischemic stroke (*n* = 19,638)	*p* value	Hemorrhagic stroke (*n* = 287)	No Hemorrhagic stroke (*n* = 20,010)	*p* value
Age (years)	58.1 (46.4–65.4)	57.5 (45.3–66.2)	0.61	57.9 (46.1–65.6)	57.5 (45.3–66.2)	0.82
Female	234 (36%)^f^	6,310 (32%)^g^	0.09	114 (40%)^h^	6430 (32%)^i^	0.01
Center volume per year	19 (8–32)	18 (9–31)	0.50	20 (9–32)	18 (9–31)	0.50
Aortic cannulation	129 (20%)	3696 (19%)	0.66	49 (17%)	3776 (19%)	0.49
Year of ECMO						
2012	20 (3%)	581 (3%)	0.35	10 (3%)	591 (3%)	0.17
2013	25 (4%)	718 (4%)		16 (6%)	727 (4%)	
2014	27 (4%)	1001 (5%)		12 (4%)	1016 (5%)	
2015	51 (8%)	1352 (7%)		15 (5%)	1388 (7%)	
2016	55 (8%)	1716 (9%)		21 (7%)	1750 (9%)	
2017	59 (9%)	2365 (12%)		24 (8%)	2400 (12%)	
2018	100 (15%)	2726 (14%)		34 (12%)	2792 (14%)	
2019	123 (19%)	3249 (17%)		55 (19%)	3317 (17%)	
2020	96 (15%)	2975 (15%)		51 (18%)	3020 (15%)	
2021	103 (16%)	2955 (15%)		49 (17%)	3009 (15%)	
Pre-ECMO ABG						
pH (2012–2016)^a^	7.25 (7.12–7.35)^f^	7.30 (7.19–7.39)^g^	0.002	7.25 (7.13–7.32)^h^	7.30 (7.19–7.39)^i^	0.004
pH (2017–2021)^b^	7.28 (7.17–7.37)^f^	7.30 (7.21–7.39)^g^	**0.0002**	7.29 (7.20–7.36)^h^	7.30 (7.21–7.39)^i^	0.04
PaO_2_ (2012–2016)^a^	95 (59–216)^f^	96 (64–197)^g^	0.35	97 (68–159)^h^	96 (64–197)^i^	0.32
PaO_2_ (2017–2021)^b^	100 (68–254)^f^	116 (74–232)^g^	0.63	107 (76–218)^h^	116 (74–233)^i^	0.25
PaCO_2_ (2012–2016)^a^	41 (31–53)^f^	40 (32.5–49.1)^g^	0.79	38 (30–45)^h^	40 (32.4–49.5)^i^	0.01
PaCO_2_ (2017–2021)^b^	41 (35–50.8)^f^	40 (33.8–48.7)^g^	0.04	39 (32–48)^h^	40 (33.9–48.8)^i^	0.60
ABG at 24-h post-cannulation						
pH (2012–2017)^c^	7.42 (7.38–7.45)^f^	7.42 (7.38–7.47)^g^	0.50	7.41 (7.36–7.45)^h^	7.42 (7.38–7.47)^i^	0.18
pH (2018–2021)^d^	7.43 (7.37–7.47)^f^	7.43 (7.38–7.47)^g^	0.29	7.43 (7.39–7.48)^h^	7.43 (7.38–7.47)^i^	0.09
PaO_2_ (2012–2017)^c^	149 (92–254)^f^	143 (94–238)^g^	0.62	157 (91–299)^h^	143 (94–238)^i^	0.15
PaO_2_ (2018–2021)^d^	132 (88–270)^f^	134 (91–222)^g^	0.08	129 (86–207)^h^	134 (91–224)^i^	0.81
PaCO_2_ (2012–2017)^c^	38 (33–41.4)^f^	38 (31–41)^g^	0.03	38.5 (33–42.8)^h^	38 (33–42)^i^	0.96
PaCO_2_ (2018–2021)^d^	38 (33.8–42)^f^	38 (33.8–42)^g^	0.66	38 (34–43)^h^	38 (33.8–42)^i^	0.84
Blood pump flow rate (2018–2021)^d^	3.84 (3.28–4.47)^f^	3.88 (3.20–4.41)^g^	0.51	3.92 (3.20–4.45)^h^	3.88 (3.20–4.41)^i^	0.49
Days on ECMO support	6.17 (3.71–10.04)	4.75 (2.71–7.67)	** < 0.0001**	6.33 (3.71–10.81)	4.79 (2.71–7.71)	** < 0.0001**
Timing of stroke (days) (2018–2021)	3.04 (1.29–6.29)^f^	N/A	N/A	3.79 (1.25–6.88)	N/A	N/A
Mortality						
90-Day mortality	427 (65%)	7123 (36%)	** < 0.0001**	206 (72%)	7344 (37%)	** < 0.0001**
30-Day mortality	411 (62%)	6339 (32%)	** < 0.0001**	195 (68%)	6555 (33%)	** < 0.0001**
ECMO complications						
Cardiac arrhythmia	129 (20%)	2112 (11%)	** < 0.0001**	52 (18%)	2189 (11%)	**0.0002**
CPB	166 (25%)	4172 (21%)	0.02	54 (19%)	4284 (21%)	0.32
Brain death (2018–2021)^e^	9 (2%)	42 (0.4%)	** < 0.0001**	7 (4%)	44 (0.4%)	** < 0.0001**
Gastrointestinal hemorrhage	48 (7%)	601 (3%)	** < 0.0001**	27 (9%)	622 (3%)	** < 0.0001**
Hemolysis (Moderate-Severe)	22 (3%)	261 (1%)	** < 0.0001**	7 (2%)	276 (1%)	0.21
Neurosurgical Intervention (2018–2021)^e^	5 (1%)	8 (0.07%)	** < 0.0001**	8 (4%)	5 (0.04%)	** < 0.0001**
Pump failure	7 (1%)	100 (0.5%)	0.10	4 (1%)	103 (0.5%)	0.10
Renal replacement therapy	237 (36%)	4509 (23%)	** < 0.0001**	94 (33%)	4652 (23%)	**0.0002**

All data are presented as *n* (%) for categorical variables, and median (interquartile range) for continuous variables

Bolded text: Statistically significant with *p* < 0.05

^a﻿^Worst” values

^b^Closest” value to ECMO start time

^c^Best” values

^d^Closest” value to post-cannulation 24-h of ECMO

^e^Used sample size of patients in years 2018–2021 as denominator

^f^Missing values for variables in the “Ischemic Stroke” group: Sex (*n* = 3), pre-ECMO pH (*n* = 175), pre-ECMO PaO_2_ (*n* = 188), pre-ECMO PaCO_2_ (*n* = 182), 24-h pH (*n* = 43), 24-h PaO_2_ (*n* = 53), 24-h PaCO_2_ (*n* = 52), 24-h blood pump flow rate (*n* = 36), timing of stroke (*n* = 9)

^g^Missing values for variables in the “No Ischemic Stroke” group: Sex (*n* = 176), pre-ECMO pH (*n* = 4995), pre-ECMO PaO_2_ (*n* = 5584), pre-ECMO PaCO_2_ (*n* = 5503), 24-h pH (*n* = 2644), 24-h PaO_2_ (*n* = 3247), 24-hr PaCO_2_ (*n* = 3207), 24-h blood pump flow rate (*n* = 1786)

^h^Missing values for variables in the “Hemorrhagic Stroke” group: pre-ECMO pH (*n* = 65), pre-ECMO PaO_2_ (*n* = 70), pre-ECMO PaCO_2_ (*n* = 68), 24-h-pH (*n* = 30), 24-h PaO_2_ (*n* = 34), 24-h PaCO_2_ (*n* = 35), 24-h blood pump flow rate (*n* = 23)

^i^Missing values for variables in the “No Hemorrhagic Stroke” group: Sex (*n* = 179), pre-ECMO pH (*n* = 5105), pre-ECMO PaO_2_ (*n* = 5702), pre-ECMO PaCO_2_ (*n* = 5617), 24-h pH (*n* = 2657), 24-h PaO_2_ (*n* = 3266), 24-h PaCO_2_ (*n* = 3224), 24-h blood pump flow rate (*n* = 1999); ABG: arterial blood gas; CPB: cardiopulmonary bypass; ECMO: extracorporeal membrane oxygenation; N/A: not applicable

### Trend of V-A ECMO associated ischemic stroke, hemorrhagic stroke, and mortality

**Fig. 1:**
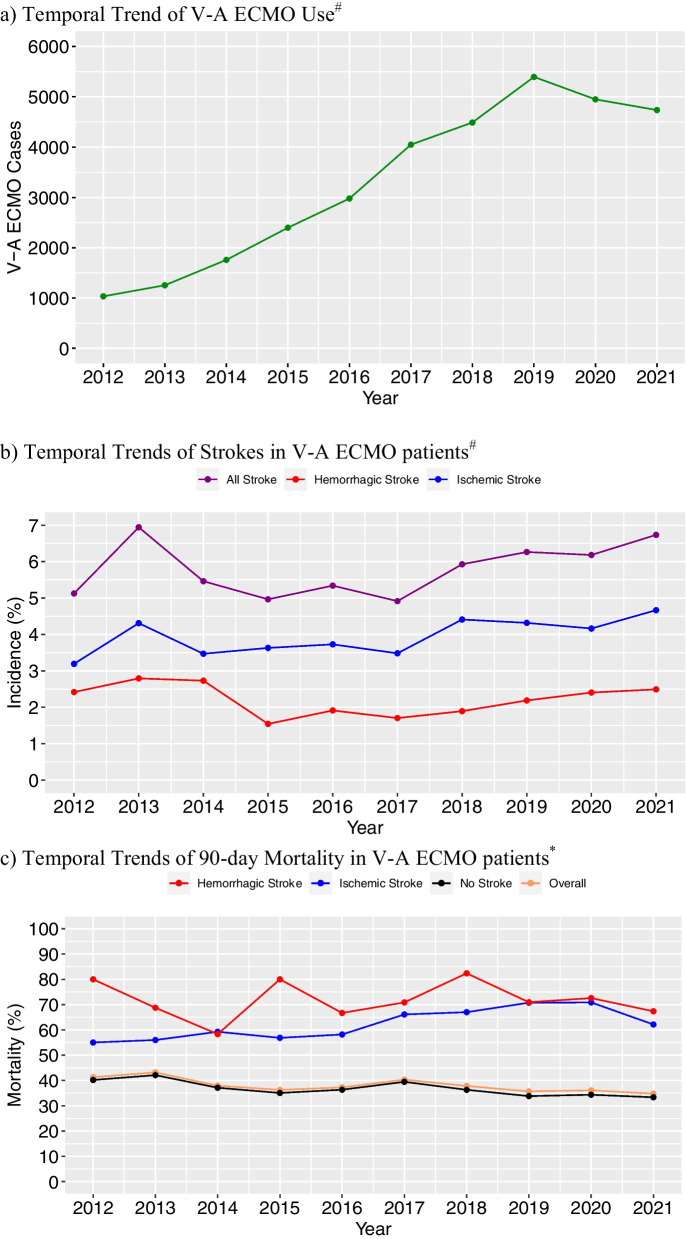
Temporal Trends of V-A ECMO, Strokes, and 90-day Mortality. **a** Temporal Trend of V-A ECMO Use^#^. **b** Temporal Trends of Strokes in V-A ECMO patients^#^. **c** Temporal Trends of 90-day Mortality in V-A ECMO patients^*^. ^#^: All 33,041 cases; ^*^: 20,297 cases with complete mortality data; V-A ECMO: venoarterial extracorporeal membrane oxygenation

Figure 1a shows the trend of V-A ECMO cases submitted to the ELSO registry database. The number of cases has generally increased during the past decade, from 1034 cases in 2012 to 4737 cases in 2021, corresponding to a roughly 1.18 times increase per year (*p* < 0.0001, Poisson regression). Figure 1b depicts the temporal trends of the incidence of ischemic and hemorrhagic strokes. The incidence of hemorrhagic stroke did not change overall between 2012 and 2021 (*p* = 0.41, Cochran-Armitage). However, the incidence of ischemic stroke increased (*p* = 0.002, Cochran-Armitage), with the number of cases increasing roughly 1.21 times per year (*p* < 0.0001, Poisson regression). Figure 1c and Additional file 2 illustrate the temporal trends of 90-day and 30-day mortality, respectively. The overall 90-day mortality declined (*p* < 0.0001, Cochran-Armitage) by 1.78% per year between 2012 and 2021 (*p* = 0.0003, Poisson regression). Similarly, 90-day mortality of patients without strokes also declined (*p* < 0.0001, Cochran-Armitage) by 2.02% per year (*p* < 0.0001, Poisson regression). In contrast, 90-day mortality of patients with hemorrhagic stroke did not significantly change over time (*p* = 0.85). While the 90-day mortality of patients with ischemic stroke showed a potential trend toward increased mortality over time, this did not reach statistical significance (*p* = 0.053).

### Association between ELSO center volume and incidence of ischemic and hemorrhagic stroke

The center volume per year for ELSO centers ranged from 1 to 84 (median 18, IQR 9–31). Multivariable logistic regression revealed that the center volume per year was not a significant factor for the incidence of ischemic or hemorrhagic stroke (Table 2). On the other hand, 24-h PaO_2_ and ΔPaCO_2_ were significantly associated with ischemic stroke incidence—for each increase of 10 mmHg in PaO_2_, the odds of ischemic stroke increased by 1.6% (*p* = 0.0006); and for each decrease of 10 mmHg in ΔPaCO_2_, the odds of ischemic stroke increased by 10.7% (*p* = 0.0009).

**Table 2:** Logistic regression of risk factors for ischemic or hemorrhagic stroke incidence^^^

	Odds ratio	95% CI	*p* value
Ischemic stroke incidence			
Age (years)	1.006	0.998–1.013	0.18
Center volume per year	1.005	0.998–1.012	0.17
Year of ECMO support			
2019^a^	0.990	0.707–1.390	0.95
2020^a^	1.042	0.741–1.467	0.81
2021^a^	1.113	0.795–1.564	0.53
Aortic cannulation	0.887	0.645–1.199	0.45
ABG related factors			
24-h PaO_2_ (mmHg)	1.002	1.001–1.002	**0.0006**
ΔPaCO_2_ (mmHg)	**0.990**	0.984–0.996	**0.0009**
24-h Blood Pump Flow Rate (L/minute)	1.071	0.941–1.219	0.30
Hemorrhagic Stroke Incidence			
Age (years)	0.998	0.987–1.010	0.80
Center volume per year	1.009	0.998–1.019	0.09
Year of ECMO Support			
2019^a^	1.385	0.828–2.366	0.22
2020^a^	1.304	0.766–2.257	0.33
2021^a^	1.384	0.816–2.389	0.23
Aortic cannulation	0.825	0.485–1.329	0.45
ABG related factors			
24-h PaO_2_ (mmHg)	0.999	0.997–1.000	0.21
ΔPaCO_2_ (mmHg)	1.000	0.990–1.011	0.98
24-h blood pump flow rate (L/minute)	0.986	0.812–1.197	0.89

Bolded text: Statistically significant with *p* < 0.05

Abbreviations: ^:12,327 patients from 2018 to 2021 with complete mortality data; ^a^Year 2018 was used as the dummy variable, as the year of ECMO support was used as a categorical variable; ΔPaCO_2_: change in arterial carbon dioxide pressure between pre-ECMO and 24-h post-cannulation; 24-h PaO_2_: 24-h post-cannulation arterial oxygen pressure; ABG: arterial blood gas; CI: confidence interval; ECMO: extracorporeal membrane oxygenation

### Association between ELSO center volume and mortality

Multivariable logistic regression revealed that lower center volume per year was significantly associated with increased 90-day mortality (*p* < 0.0001). Additionally, the complications of ischemic stroke and hemorrhagic stroke significantly increased mortality (*p* < 0.0001 for both). Other significantly associated variables included older age, higher 24-h PaO_2_, higher 24-h blood pump flow rate, longer duration of ECMO, and complications of gastrointestinal hemorrhage and renal replacement therapy (Table 3, Additional file 3). Brain death occurred in 51 patients (0.4%), however, could not be included in the logistic regression model, given that no patients with brain death (*n* = 0) had the outcome of survival. Given that both the 24-h PaO_2_ and the 24-h blood pump flow rate were significantly associated with mortality, a Pearson’s correlation coefficient was calculated, which did not reveal a strong linear correlation (R^2^ = 0.0016) (Additional file 4).

**Table 3:** Logistic regression of risk factors for 90-day mortality^^^

	Odds ratio	95% CI	*p* value
Age (years)	1.036	1.032–1.039	** < 0.0001**
Center volume per year	0.990	0.987–0.993	** < 0.0001**
Year of ECMO support			
2019^a^	0.872	0.760–1.002	0.05
2020^a^	0.929	0.807–1.068	0.30
2021^a^	0.862	0.748–0.903	**0.04**
Aortic cannulation	0.911	0.804–1.052	0.18
ABG related factors			
24-h PaO_2_ (mmHg)	1.001	1.001–1.002	** < 0.0001**
ΔPaCO_2_ (mmHg)	1.000	0.997–1.003	0.79
24-h blood pump flow rate (L/minute)	1.077	1.020–1.138	**0.008**
ECMO duration (days)	1.019	1.011–1.027	** < 0.0001**
Neurological complications			
Cardiac arrhythmia	1.075	0.925–1.249	0.34
Cardiopulmonary bypass	1.006	0.890–1.136	0.93
Gastrointestinal hemorrhage	1.722	1.328–2.239	** < 0.0001**
Hemolysis (moderate-to-severe)	1.315	0.966–1.785	0.08
Hemorrhagic stroke	2.918	1.961–4.396	** < 0.0001**
Ischemic stroke	2.987	2.299–43.902	** < 0.0001**
Neurosurgical intervention	1.846	0.459–8.014	0.39
Pump failure	1.154	0.569–2.308	0.69
Renal replacement therapy	2.105	1.885–2.350	** < 0.0001**

Bolded text: Statistically significant with *p* < 0.05

Abbreviations: ^^^:12,327 patients from 2018 to 2021 with complete mortality data; ^a^: Year 2018 was used as the dummy variable, as the year of ECMO support was used as a categorical variable; ΔPaCO_2_: change in arterial carbon dioxide pressure between pre-ECMO and 24-h post-cannulation; 24-h PaO_2_: 24-h post-cannulation arterial oxygen pressure; ABG: arterial blood gas; CI: confidence interval; ECMO: extracorporeal membrane oxygenation

### Ischemic and hemorrhage stroke as risk factors for mortality

**Fig. 2:**
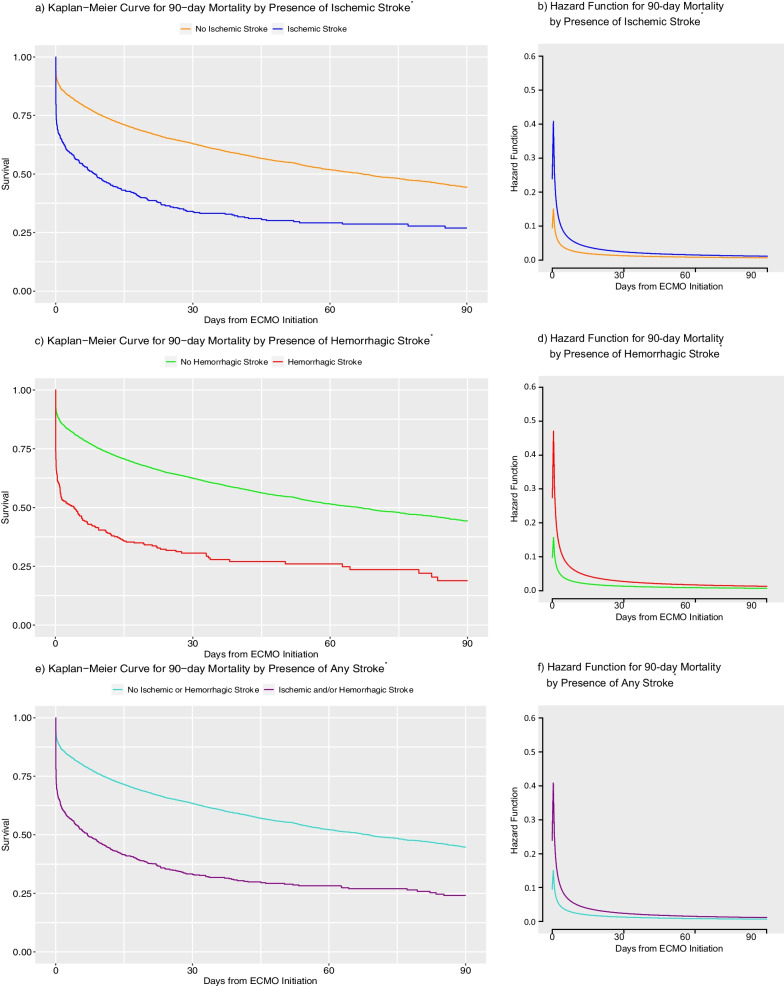
Kaplan–Meier Curves and Hazard Functions for 90-day Survival by the Presence of Strokes. Abbreviations: ^*^: 20,297 cases with complete mortality data; ECMO: extracorporeal membrane oxygenation

Figure 2 and Additional file 5 show the overall survival curves and hazard functions of V-A ECMO patients with or without strokes. Median 90-day survival times were significantly different between patients who developed strokes and those who did not: 8.54 vs 67.17 days in the presence or absence of ischemic stroke, respectively (*p* < 0.0001, log-rank test); 4.00 vs 66.20 days in the presence or absence of hemorrhagic stroke, respectively (*p* < 0.0001, log-rank test); and 7.00 vs 68.80 days in the presence or absence of any stroke, respectively (*p* < 0.0001, log-rank test).

The multivariable logistic regression models for 90-day and 30-day mortality revealed numerous, significantly associated variables (Table 3, Additional file 3). In particular, the odds of 90-day mortality were higher for patients with ischemic stroke than those without (odds ratio = 2.987, *p* < 0.0001, logistic regression). This was also similar to the case of 90-day mortality of patients with hemorrhagic stroke compared to those without (odds ratio = 2.918, *p* < 0.0001, logistic regression).

## Discussion

This study is the largest to date that assesses the associations of mortality and strokes in the context of other ECMO-related variables, as well as the trends of stroke incidence over 10 years in V-A ECMO. The ELSO registry database has grown markedly, with the number of reported cases increasing nearly fivefold over the past decade, continuing the trend identified in the ELSO study of V-A ECMO from 1992 to 2013 [2]. Encouragingly, the overall 90-day and 30-day mortality rate of V-A ECMO patients have steadily down-trended over time (Fig. 1, Additional file 2). This is also consistent with the survival trends of ECMO patients in the ELSO database since its inception in 1989, which has steadily risen over time from < 1% survival rate [7]. However, the survival for patients who developed ischemic and/or hemorrhagic strokes has not improved. While the incidence of ischemic stroke appears to have mildly increased over time, this may be in the setting of more recognition and vigilance regarding ECMO-related complications, leading to more detection of ischemic strokes, rather than worsening ECMO management. For example, a study conducted at Johns Hopkins Hospital demonstrated that a standardized neuromonitoring protocol for ECMO patients led to higher detection of acute brain injury, yet improved patient outcomes [1]. Alternatively, the increasing incidence of strokes as a complication of V-A ECMO over time may be due to V-A ECMO being offered to a progressively wider patient population, including those who may be more critically ill than in prior years, as ECMO use and practice have evolved over time with more experience and standardized ECMO care. Overall, the incidences of ischemic and hemorrhagic strokes as complications of V-A ECMO were 3.2% and 1.4%, respectively. For comparison, a prior study of V-V ECMO patients in the ELSO registry revealed that the incidences of ischemic and hemorrhagic strokes were 1.4% and 3.1%, respectively [3]. As expected, the incidence of ischemic stroke was higher in V-A ECMO than in V-V ECMO. The fact that the incidence of hemorrhagic stroke was higher in V-V ECMO may be attributed to the fact that cannulation of the internal jugular vein (especially double-lumen cannulation) is more commonly performed, which may lead to complications of cerebral venous sinus thrombosis and/or venous hypertension that can further progress into hemorrhagic strokes [8]. Another potential factor is that the duration of ECMO is longer in V-V EMCO compared to V-A ECMO, thereby increasing the exposure time.

Risk factors associated with on-ECMO complications of ischemic stroke in the logistic regression model included 24-h PaO_2_ and ΔPaCO_2_ (Table 2). These findings are consistent with a prior ELSO study of V-A ECMO from a slightly older timeframe, which identified that large reductions in PaCO_2_ over 24-h were associated with increased neurological complications [9]. Another recent ELSO study, albeit regarding the ECPR patient population, also showed that early severe hyperoxia during ECMO is a significant risk factor for complications of acute brain injury [10]. However, center volume per year was not associated with incidence of ischemic or hemorrhagic stroke. This may be related to the fact that there is no standardized protocol for monitoring patients for on-ECMO complications of strokes, which is made especially difficult due to limitations in performing reliable physical exams in the setting of sedating medications. Further studies are needed to identify the optimal timing of neuro-imaging to detect strokes during ECMO.

In contrast, ELSO centers with higher annual case volumes generally had lower mortality, such that for each 10 additional cases per year performed at a given ELSO center, the 90-day mortality reduced by 9.7%. This finding is similar to other ECMO studies of different patient populations (ECPR, ECMO for respiratory failure, and ECMO for cardiogenic shock), which also identified that mortality was lower at centers with higher annual case volumes [11–13]. This association is likely, at least in part, due to ECMO centers with higher annual case volumes having more established infrastructure, experience, and knowledge of ECMO therapy, thereby achieving greater success rates.

Additionally, the presence of ischemic and/or hemorrhagic stroke was significantly associated with increased 90-day and 30-day mortality (Fig. 2, Additional file 5). The hazard of death was highest in the first week of ECMO support, by which time 82% of all ischemic strokes and 78% of all hemorrhagic strokes had occurred, then plateaued in the subsequent weeks. Hazards were even higher for patients who developed strokes early on as a complication of ECMO compared to those who did not. Prior literature evaluating the associations between mortality and strokes are limited, both in quantity and the mostly single-center nature of the studies. Overall, these studies have shown mixed results, with some reporting hemorrhagic stroke, but not ischemic stroke, as a risk factor for increased mortality during ECMO support [14, 15], while others also reporting ischemic stroke to be a risk factor [16–19]. In this context, our multi-center study contributes further evidence that both ischemic and hemorrhagic strokes are associated with increased 90-day and 30-day mortality.

The logistic regression model identified numerous other risk factors that are associated with higher mortality (Table 3, Additional file 3). Patients who require longer ECMO support are likely more critically ill and more prone to developing on-ECMO complications, which can lead to poorer prognosis. The positive associations between mortality and the risk factors of older age, longer ECMO duration, higher PaO_2_, and renal replacement therapy are also consistent with prior studies [4, 9, 20]. Additionally, the fact that gastrointestinal hemorrhage, renal replacement therapy, and brain death are associated with both incidence of ischemic stroke and mortality suggests that these on-ECMO complications likely represent a diffuse, systemic process that collectively contributes to increased mortality (Table 1). Finally, in contrast to the incidence of ischemic stroke, mortality was associated with higher 24-h blood pump flow rate. However, there was no significant linear correlation between higher 24-h blood pump flow rate and 24-h PaO_2_ (Additional file 4). This suggests that while hyperoxia can be detrimental during ECMO by increasing the risk of both ischemic strokes and mortality, these relations cannot be attributed to the blood pump flow rates alone. The strong association between higher 24-h blood pump flow rate and mortality may be representative of patients who lack native cardiac output and thus are more critically ill.

### Limitations

This study has several limitations. First, there were missing and/or unavailable data in the ELSO database, including the size and severity of strokes during ECMO support, which could not be accounted for in the analyses. Nevertheless, the major advantage of the ELSO registry data is its large sample size that allows for higher statistical power, hundreds of available variables, and the multi-institutional nature that allows for generalizability of the study. Second, while the center volume analysis focused on annual case volume, this does not account for the number of years that the centers have been performing ECMO support. However, total center volume registered in the ELSO registry was not chosen as the variable, as the ECMO centers may have operating ECMO programs prior to joining ELSO, thereby not reflecting the true total center experience. Third, the ECMO protocols of the numerous ELSO centers are not necessarily standardized, which may lead to some differences in diagnoses of complications, such as how and when strokes were diagnosed and/or management decisions, such as when neurosurgical interventions were performed. Given that these medical decisions were made based on best clinical judgment and necessity, these differences were considered to be relatively minor overall. Finally, withdrawal of life sustaining therapies was not accounted for in the database or analysis, which often limits the outcomes studies of critically ill patient populations.

## Conclusions

Over the past decade, while the incidence of ischemic stroke in V-A ECMO patients has progressively increased, 90-day mortality has decreased, possibly explained by the increased awareness, detection, and management of on-ECMO complications. Higher 24-h PaO_2_ and greater decrease in PaCO_2_ from pre-ECMO to 24-h values were associated with greater ischemic stroke incidence, while higher annual center volume was not. On-ECMO complications of ischemic/hemorrhagic strokes, gastrointestinal hemorrhage, and renal replacement, as well as higher 24-h PaO_2_ and longer ECMO duration, were associated with increased 90-day mortality. In contrast, higher annual center volume was significantly associated with lower 90-day mortality. Hazard of death was highest in the first several days of V-A ECMO.

### Supplementary Information

**Additional file 1.** Flowchart of patient selection.

**Additional file 2.** Temporal trends of 30-day mortality in V-A ECMO patients.

**Additional file 3.** Logistic regression of risk factors for 30-day mortality.

**Additional file 4.** Correlation between 24-hour PaO_2_ and 24-hour blood pump flow rate.

**Additional file 5.** Kaplan–Meier curves and hazard functions for 30-day survival by the presence of strokes.

## Data Availability

The data that support the findings of this study are available from the Extracorporeal Life Support Organization (ELSO), but restrictions apply to the availability of these data, which were used under license for the current study, and so are not publicly available. Data are, however, available from the authors upon reasonable request and with permission of ELSO.

## Abbreviations

ΔPaCO_2_: Change in PaCO_2_ between pre-ECMO and post-cannulation 24-h values
CT: Computed tomography
ECMO: Extracorporeal membrane oxygenation
ECPR: Extracorporeal cardiopulmonary resuscitation
ELSO: Extracorporeal Life Support Organization
ICD-9: International Classification of Diseases, 9th Revision
ICD-10: International Classification of Diseases, 10th Revision
IQR: Interquartile range
MRI: Magnetic resonance imaging
PaO_2_: Arterial oxygen pressure
PaCO_2_: Arterial carbon dioxide pressure
V-A: Venoarterial
