# Supplementary material for: Epidemiology of ischemic stroke and hemorrhagic stroke in venoarterial extracorporeal membrane oxygenation
Source: Crit Care. 2023 Nov 9;27:433. doi: 10.1186/s13054-023-04707-z (PMC10633935; doi:10.1186/s13054-023-04707-z)
Supplement: Supplementary file 1 — Additional file 1. Flowchart of patient selection. [file 13054_2023_4707_MOESM1_ESM.docx]

Additional File 1: Flowchart of Patient Selection

Patient records in ELSO registry data of V-A ECMO in 2012-2021

n=34,734

Patients with V-A ECMO only

(Figures 1a, 1b)

n=33,041

Patients whose V-A ECMO was later converted to a different mode

n=1,693

Patients with uncertain discharge status

n=2,552

Patients with uncertain time-to-discharge/death

n=10,192

Patients from 2012-2017

n=7,970

Patients with complete mortality data

(Figures 1c, 2a-2f)

n=20,297

Patients with complete ABG data

(Logistic regression models)

n=12,327

Abbreviations: ABG: arterial blood gas; ELSO: Extracorporeal Life Support Organization; V-A ECMO: venoarterial extracorporeal membrane oxygenation
