# Supplementary material for: Epidemiology of ischemic stroke and hemorrhagic stroke in venoarterial extracorporeal membrane oxygenation
Source: Crit Care. 2023 Nov 9;27:433. doi: 10.1186/s13054-023-04707-z (PMC10633935; doi:10.1186/s13054-023-04707-z)
Supplement: Supplementary file 2 — Additional file 2. Temporal trends of 30-day mortality in V-A ECMO patients. [file 13054_2023_4707_MOESM2_ESM.docx]

Additional File 2: Temporal Trends of 30-day Mortality in V-A ECMO Patients^*^

Abbreviations: ^*^: 20,297 cases with complete mortality data; V-A ECMO: venoarterial extracorporeal membrane oxygenation
