# Supplementary material for: Epidemiology of ischemic stroke and hemorrhagic stroke in venoarterial extracorporeal membrane oxygenation
Source: Crit Care. 2023 Nov 9;27:433. doi: 10.1186/s13054-023-04707-z (PMC10633935; doi:10.1186/s13054-023-04707-z)
Supplement: Supplementary file 3 — Additional file 3. Logistic regression of risk factors for 30-day mortality. [file 13054_2023_4707_MOESM3_ESM.docx]

Additional File 3: Logistic Regression of Risk Factors for 30-day Mortality^^^

|  | **Odds ratio** | **95% CI** | **p-value** |
| --- | --- | --- | --- |
| **Age (years)** | **1.035** | 1.031-1.039 | **<0.0001** |
| **Center Volume Per Year** | **0.988** | 0.985-0.991 | **<0.0001** |
| **Year of ECMO Support** | | | |
| **2019*^a^*** | **0.868** | **0.754-0.9995** | **0.049** |
| 2020***^a^*** | 0.912 | 0.791-1.052 | 0.20 |
| **2021*^a^*** | **0.848** | **0.734-0.980** | **0.03** |
| Aortic Cannulation | 0.896 | 0.779-1.030 | 0.12 |
| ABG related factors | | | |
| **24-hour PaO_2_ (mmHg)** | **1.001** | **1.001-1.001** | **<0.0001** |
| ΔPaCO_2_ (mmHg) | 0.998 | 0.995-1.001 | 0.25 |
| **24-hour Blood Pump Flow Rate (L/minute)** | **1.088** | **1.029-1.030** | **0.003** |
| **ECMO Duration (days)** | **1.011** | **1.003-1.019** | **0.006** |
| **Neurological Complications** | | | |
| Cardiac Arrhythmia | 1.064 | 0.914-1.238 | 0.42 |
| Cardiopulmonary Bypass | 0.970 | 0.856-1.098 | 0.63 |
| **Gastrointestinal Hemorrhage** | **1.693** | **1.308-2.192** | **<0.0001** |
| Hemolysis (Moderate-Severe) | 1.303 | 0.954-1.770 | 0.09 |
| **Hemorrhagic Stroke** | **2.509** | **1.703-3.718** | **<0.0001** |
| **Ischemic Stroke** | **3.355** | **2.593-4.362** | **<0.0001** |
| Neurosurgical Intervention | 2.234 | 0.560-9.666 | 0.26 |
| Pump Failure | 0.958 | 0.459-1.930 | 0.91 |
| **Renal Replacement Therapy** | **2.002** | **1.791-2.238** | **<0.0001** |

Abbreviations: ^^^:12,327 patients from 2018-2021 with complete mortality data; ***^a^***: Year 2018 was used as the dummy variable, as the year of ECMO support was used as a categorical variable; ΔPaCO_2_: change in arterial carbon dioxide pressure between pre-ECMO and 24-hour post-cannulation; 24-hour PaO_2_: 24-hour post-cannulation arterial oxygen pressure; ABG: arterial blood gas; CI: confidence interval; ECMO: extracorporeal membrane oxygenation
