# Supplementary material for: Epidemiology of ischemic stroke and hemorrhagic stroke in venoarterial extracorporeal membrane oxygenation
Source: Crit Care. 2023 Nov 9;27:433. doi: 10.1186/s13054-023-04707-z (PMC10633935; doi:10.1186/s13054-023-04707-z)
Supplement: Supplementary file 4 — Additional file 4. Correlation between 24-hour PaO2 and 24-hour blood pump flow rate. [file 13054_2023_4707_MOESM4_ESM.docx]

Additional File 4: Correlation between 24-hour PaO_2_ and 24-hour Blood Pump Flow Rate

Abbreviation: PaO_2_: arterial oxygen pressure
