# Supplementary material for: Epidemiology of ischemic stroke and hemorrhagic stroke in venoarterial extracorporeal membrane oxygenation
Source: Crit Care. 2023 Nov 9;27:433. doi: 10.1186/s13054-023-04707-z (PMC10633935; doi:10.1186/s13054-023-04707-z)
Supplement: Supplementary file 5 — Additional file 5. Kaplan–Meier curves and hazard functions for 30-day survival by the presence of strokes. [file 13054_2023_4707_MOESM5_ESM.docx]

Additional File 5: Kaplan-Meier Curves and Hazard Functions for 30-day Survival by Presence of Strokes

b) Hazard Function for 30-day Mortality

by Presence of Ischemic Stroke^*^

d) Hazard Function for 30-day Mortality

by Presence of Hemorrhagic Stroke^*^

f) Hazard Function for 30-day Mortality

by Presence of Any Stroke^*^

Days from ECMO Initiation

Hazard Function

0 15 30

0.0

0.1

0.2

0.3

0.4

0.5

0.6

Days from ECMO Initiation

0 15 30

Days from ECMO Initiation

0 15 30

Hazard Function

0.0

0.1

0.2

0.3

0.4

0.5

0.6

Hazard Function

0.0

0.1

0.2

0.3

0.4

0.5

0.6

Abbreviations: ^*^: 20,297 cases with complete mortality data; ECMO: extracorporeal membrane oxygenation

Abbreviations: ECMO: extracorporeal membrane oxygenation
